# Spatial and temporal characterization of the rich fraction of plastid DNA present in the nuclear genome of *Moringa oleifera* reveals unanticipated complexity in NUPTs´ formation

**DOI:** 10.1186/s12864-024-09979-5

**Published:** 2024-01-15

**Authors:** Juan Pablo Marczuk-Rojas, Angélica María Álamo-Sierra, Antonio Salmerón, Alfredo Alcayde, Viktor Isanbaev, Lorenzo Carretero-Paulet

**Affiliations:** 1 Department of Biology and Geology, University of Almería, Ctra. Sacramento s/n, 04120 Almería, Spain; 2 “Pabellón de Historia Natural-Centro de Investigación de Colecciones Científicas de la Universidad de Almería” (PHN-CECOUAL), University of Almería, Ctra. Sacramento s/n, Almería, 04120 Spain; 3 Department of Mathematics, University of Almería, Ctra. Sacramento s/n, 04120 Almería, Spain; 4 Department of Engineering, University of Almería, Ctra. Sacramento s/n, 04120 Almería, Spain

**Keywords:** Moringa, NUPTs, Plastid DNA, Chloroplast, Genome Evolution

## Abstract

**Background:**

Beyond the massive amounts of DNA and genes transferred from the protoorganelle genome to the nucleus during the endosymbiotic event that gave rise to the plastids, stretches of plastid DNA of varying size are still being copied and relocated to the nuclear genome in a process that is ongoing and does not result in the concomitant shrinking of the plastid genome. As a result, plant nuclear genomes feature small, but variable, fraction of their genomes of plastid origin, the so-called nuclear plastid DNA sequences (NUPTs). However, the mechanisms underlying the origin and fixation of NUPTs are not yet fully elucidated and research on the topic has been mostly focused on a limited number of species and of plastid DNA.

**Results:**

Here, we leveraged a chromosome-scale version of the genome of the orphan crop *Moringa oleifera*, which features the largest fraction of plastid DNA in any plant nuclear genome known so far, to gain insights into the mechanisms of origin of NUPTs. For this purpose, we examined the chromosomal distribution and arrangement of NUPTs, we explicitly modeled and tested the correlation between their age and size distribution, we characterized their sites of origin at the chloroplast genome and their sites of insertion at the nuclear one, as well as we investigated their arrangement in clusters. We found a bimodal distribution of NUPT relative ages, which implies NUPTs in moringa were formed through two separate events. Furthermore, NUPTs from every event showed markedly distinctive features, suggesting they originated through distinct mechanisms.

**Conclusions:**

Our results reveal an unanticipated complexity of the mechanisms at the origin of NUPTs and of the evolutionary forces behind their fixation and highlight moringa species as an exceptional model to assess the impact of plastid DNA in the evolution of the architecture and function of plant nuclear genomes.

**Supplementary Information:**

The online version contains supplementary material available at 10.1186/s12864-024-09979-5.

## Background

Nearly all plants contain a small, but significant, fraction of their nuclear genomes composed of DNA sequences derived from their chloroplasts [1]; these nuclear integrants of plastid DNA are commonly known as nuclear plastid DNA sequences (NUPTs) [2] The process of NUPTs´ formation has been commonly associated to the process by which most genes present in the bacterial ancestor of plastids were transferred to the nuclear genome and their products eventually retargeted to their ancestral compartment after the endosymbiotic event that gave rise to the chloroplast organelle. However, whereas the latter entails the loss of vast amounts of DNA with the subsequent reduction of its size and the transfer of most of the genes originally present in the protoorganelle organism to the nuclear genome [3, 4], the former involves the copy of stretches of DNA from the chloroplast genome. Even though most NUPTs are less than 1 kb in length, NUPTs of recent origin spanning the whole chloroplast chromosome have been detected in *Oryza sativa* (rice) and *Populus trichocarpa* [5, 6], and did not result in the shrinking of the plastid genome.

Although the process of NUPTs’ formation is still poorly understood, it is expected to involve the following sequence of events. First, the duplication of a stretch of DNA present in the chloroplast genome. Second, the lysis of chloroplast organelle membranes to allow the leakage of duplicated plastid DNA. Third, the import to the nucleus of the leaked plastid DNA. Fourth, the integration of plastid DNA into the nuclear genome. At present, no mechanism has been formally proposed to explain the recurrent duplication of stretches of plastid DNA of varying sizes that are at the origin of NUPTs. The biological mechanisms involved in the leakage of plastid DNA to the cytoplasm and its subsequent import by the nucleus are not yet completely elucidated either, although gametogenesis and cell stress (especially pollen development and mild heat stress, respectively) have been reported to induce the disruption of chloroplast organelle membranes [2, 7–10]. It has been also suggested that certain kinds of stresses, such as ionizing radiation and pathogen infections, may, not only trigger the leakage of plastid DNA to the nucleocytosolic compartment, but also favor its integration into the nuclear genome [11]. The molecular mechanisms of NUPTs´ integration into the nuclear genome are not fully described either, but they are probably diverse and generally involve double-stranded breaks (DSBs) and DNA damage and thus are potentially mutagenic. For example, it has been hypothesized that NUPTs´ integration is mediated by non-homologous end joining (NHEJ) during DSB repair events [12–14], most NUPTs are expected to be rapidly fragmented and shuffled away through transpositions and genome arrangements and, eventually, purged from the nuclear genome [15–17]. As a consequence, the distribution of NUPTs by age should follow an exponential distribution, indicating a continuous rate of NUPTs’ formation and decay throughout time [15]. Although such a pattern has been suggested for rice, *Medicago truncatula*, *P. trichocarpa* and *Zea mays* [15, 17, 18], different patterns have been observed in other species such as Arabidopsis, *Carica papaya*, *Fragaria vesca*, *Moringa oleifera* (moringa) and *Vitis vinifera* [17–19]. A second consequence is the expected positive correlation between NUPTs’ size and age, an observation that has been suggested for several species, despite not being explicitly tested statistically [7, 16, 17, 20].

Indeed, the fraction of nuclear genomes occupied by NUPTs varies enormously among species and even within different populations of the same species [5, 21, 22]. Most species showed around 0.1% of plastid DNA in their nuclear genome, with very few showing more than 1% [1] These large variations in the fraction of nuclear genomes occupied by NUPTs raise the question of what evolutionary forces may lie behind the fixation of variable fractions of plastid DNA in plant nuclear genomes. However, previous studies on the mechanisms of origin and evolutionary fate of NUPTs were mostly focused on a limited number of species and involved a reduced number of NUPTs. A more detailed picture will certainly benefit from a larger number of NUPTs and a higher fraction of the nuclear genome occupied by plastid DNA.

So far, the largest fraction of DNA of plastid origin found in any plant nuclear genome (4.71%) has been detected in the orphan crop moringa [19]. In the present study, we leveraged a recent chromosome-scale version of the moringa genome [23] to examine the spatial distribution and arrangement in clusters of NUPTs, to explicitly model and test the correlation between their age and size distribution, to characterize their origin within the chloroplast genome and their sites of insertion at the nuclear one, as well as to investigate their arrangement in clusters. Our results reveal an unanticipated complexity of the mechanisms at the origin of NUPTs as well as of the evolutionary forces behind their fixation.

## Results

### Widespread distribution of NUPTs in the moringa nuclear genome

In order to detect NUPTs present in the moringa nuclear genome, a chromosome-scale assembly of the moringa genome, AOCCv2 [23], was scanned using BLASTN and the moringa chloroplast genome sequence (NCBI RefSeq number: NC_041432.1) [24] as query, resulting in 13,901 total alignments. We visually inspected the alignments and detected a significant fraction of them (8657; 62.28%) arising from two specific regions of the chloroplast genome. Those two regions were 200 bp and 350 bp in length and were essentially composed by As and Ts (Additional File 1), thus likely corresponding to low complexity regions, which are known to result in spurious alignments not reflecting true homology but artifacts. Indeed, BLASTN searches on NCBI databases using those two regions as queries resulted in matches to seemingly unrelated genomes with high percent of identity, indicating they probably correspond to artifacts (results not shown). Therefore, we reran BLASTN with the -dust option turned on in order to mask alignments resulting from low complexity regions. 5203 NUPTs were now detected, which were confidently defined as NUPTs in our analysis (Supplemental Table S1). 11 out of the 14 chromosomes hosted more than 100 NUPTs (ranging from 118 to 1072) and seven chromosomes plus one scaffold contained NUPTs summing up above 160,600 bp (i.e., the size of the moringa chloroplast genome) (Supplemental Table S1).

The total aligned region between the chloroplast genome and the nuclear genome, i.e., the total region of the nuclear genome occupied by NUPTs, summed up a total of 9,781,275 bp, which represents a 4.14% of the size of the nuclear genome assembly, close to estimations obtained with previous versions of the genome [25–27] (Table 1). After correcting for redundancy in BLASTN hits resulting from Inverted Repeat (IR) regions of the moringa chloroplast genome (1272), the fraction of the moringa nuclear genome corresponding to NUPTs was of 3.29%, again pretty similar to estimations obtained with the three other versions of the moringa genome [25–27] (Table 1), and further supporting these results were not due to genome assembly errors.

**Table 1:** Summary of the moringa nuclear genome versions used in this study

Nuclear genome version	Total fraction of plastid DNA (%)	Total fraction of plastid DNA after removing redundant NUPTs (%)	Reference
AOCC v2	4.14	3.29	[23]
Shyamli, et al., 2021 [27]	4.73	3.81	[27]
AOCC v1	4.25	3.28	[26]
Tian, et al., 2015 [25]	4.19	3.12	[25]

The fraction of plastid DNA detected in each version, before and after correcting for redundant NUPTs, is also indicated

### Most NUPTs in moringa originated through two distinct formation episodes separated in time

**Fig. 1:**
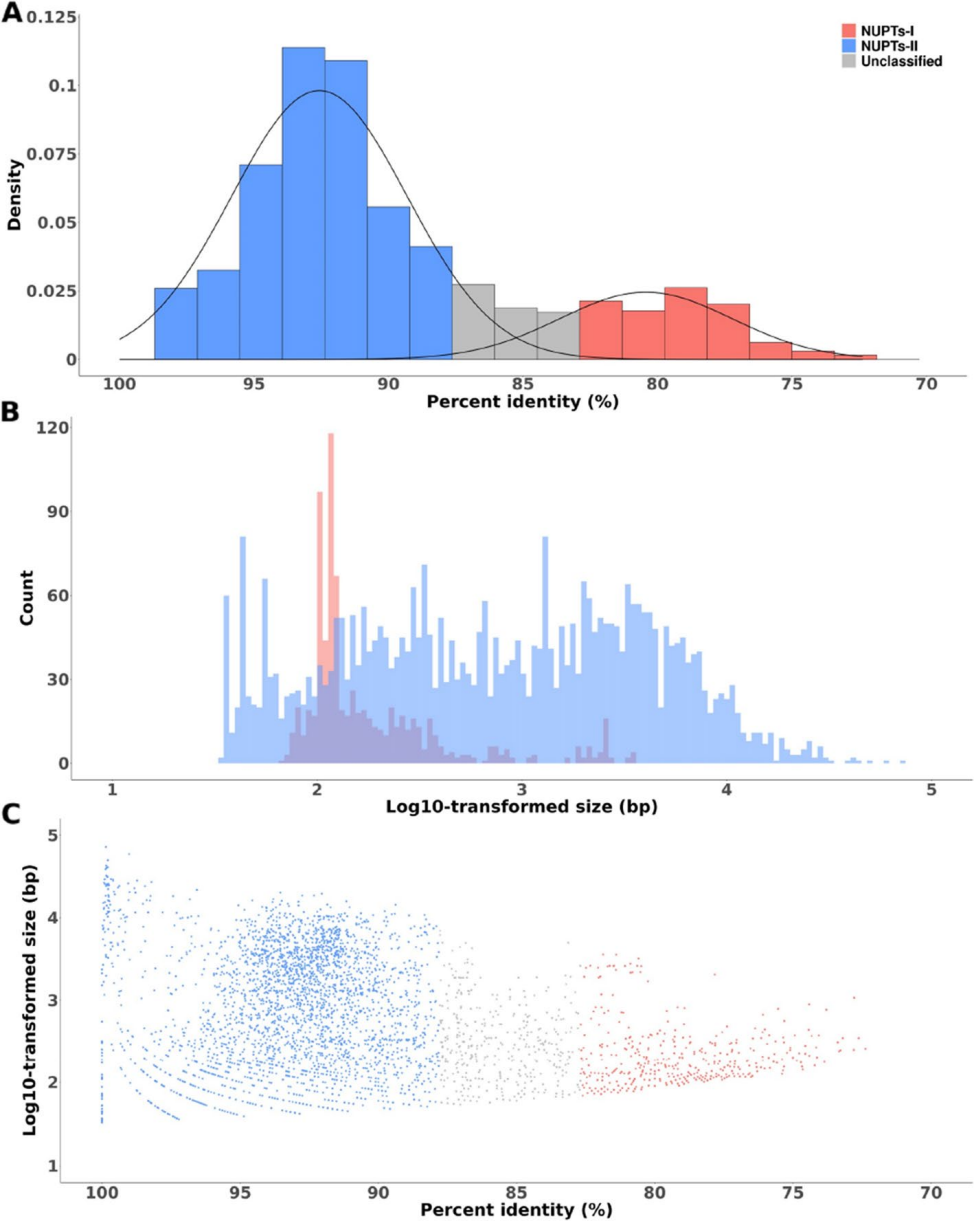
Modeling the distribution of percent identity and size of moringa NUPTs. **A** histogram of the distribution of NUPTs percent identity values. The two density plots resulting from fitting Gaussian mixture models, putatively corresponding to distinct events of NUPTs´ formation (I and II), are shown. **B** Histogram of the distribution of NUPTs size values partitioned by formation event. **C** scatterplot of percent identity versus sizes of moringa NUPTs partitioned by formation event. For an easier visualization, NUPT size values have been log 10-transformed

In order to gain insights on the timing of plastid DNA acquisition by the moringa nuclear genome, we examined the relative age distribution of NUPTs using the percent identity of the corresponding BLASTN hits as a proxy of evolutionary time. Assuming the mutation rate is proportional to evolutionary time*, i. e*., the molecular clock hypothesis holds, the lower the percent identity, the older the NUPTs. Percent identity of BLASTN hits ranges between 72.37 and 100% and shows an apparent bimodal distribution (Fig. 1A). Indeed, when Gaussian mixture models were fitted to the corresponding density curves, two clear peaks, centered around 79.05 and 93.1%, respectively, were detected (Fig. 1A). According to the posterior probabilities of assigning a NUPT to either one or another peak, using a threshold of 95%, 776 NUPTs (14.91% of the total) summing up a total of 253,096 bp (2.59% of the total) belonged to the older peak (from now on Episode I, or NUPTs-I), while 3855 NUPTs (74.09% of the total) summing up a total of 9,189,682 bp (93.95% of the total) belonged to the younger peak (from now on Episode II or NUPTs-II). The rest of NUPTs (572, summing up a total of 338,497 bp, i.e., 3.46% of the total) were not confidently assigned to either one or the other peak. Taking as a whole, these results support two main episodic formation events at the origin of most NUPTs.

Next, we examined the size distribution of NUPTs, partitioned by each of the retrieved episodes. While NUPTs-I ranged in size from 69 to 3591 bp, NUPTs-II ranged from 33 to 71,935 bp (Fig. 1B). Both followed a non-normal right-skewed unimodal distribution (Fig. 1B), with a mean and a median size of 326.2 and 127 or 2384 and 778 bp for NUPTs-I and NUPTs-II, respectively.

From studies in rice and other plant species, it had been suggested an apparent positive correlation between size and sequence identity of NUPTs, i.e., larger NUPTs tend to be more conserved at the sequence level. This observation can be interpreted as young, larger conserved NUPTs declining and fragmenting over time, and eventually being purged from the genome [7, 15–17, 20, 28]. To test whether this observation also applied to moringa NUPTs, we studied the correlation between size and sequence identity by means of two different tests appropriate for not-normally distributed data, again partitioned by every episode detected (Fig. 1C and Table 2). Interestingly, while for younger NUPTs from episode II size negatively correlated with sequence identity in both tests (Table 2), no significant correlation was found for NUPTs-I (Table 2), suggesting different mechanisms might have been at the origin of NUPTs from every episode and / or, once integrated, they might also have followed different evolutionary trajectories.

**Table 2:** Correlation analysis between NUPTs’ sequence identity and size by NUPTs’ formation event

Method	Correlation coefficient	*P*	Episode
Kendall’s rank correlation tau	−0.05	0.06	I
Spearman’s rank correlation rho	−0.03	0.42	I
Kendall’s rank correlation tau	−0.05	6.72 × 10^−7^	II
Spearman’s rank correlation rho	− 0.07	6.67 × 10^−7^	II

To provide further support to the accuracy of the obtained results and discard their origin through genome assembly errors, we repeated all the analysis using the three previously published versions of the moringa nuclear genome assembly available [25–27]. In each case, when fitting Gaussian mixture models to each distribution of percent identities, the two main peaks could be similarly retrieved (Supplemental Fig. S1 and Supplemental Table S2). Negative correlations between size and sequence identity were also similarly retrieved for NUPTs-II (Supplemental Table 3), while not significant or only marginally significant positive correlation was found for NUPTs-I.

We found 61 NUPTs, 51 of them not redundant, spanning a total of 14,177 bp, showing 100% identity with the chloroplast genome. These NUPTs might not represent a real biological phenomenon but be the result of a misassembly that erroneously incorporated plastid regions into the nuclear genome sequence. In order to discard this possibility, we sampled the sequences from six representative NUPTs showing 100% identity and various sizes plus 100 bp of their flanking regions in the nuclear genome and scanned for their occurrence in the three additional versions of the moringa nuclear genome available. As revealed by the corresponding multiple sequence alignments, the six NUPTs plus flanking regions selected could be identically retrieved in at least one of the remaining three genome versions (Additional files 2, 3, 4, 5, 6 and 7), further validating our findings.

### Characterization of the differential distribution of NUPTs´ insertion sites in the moringa nuclear genome

**Fig. 2:**
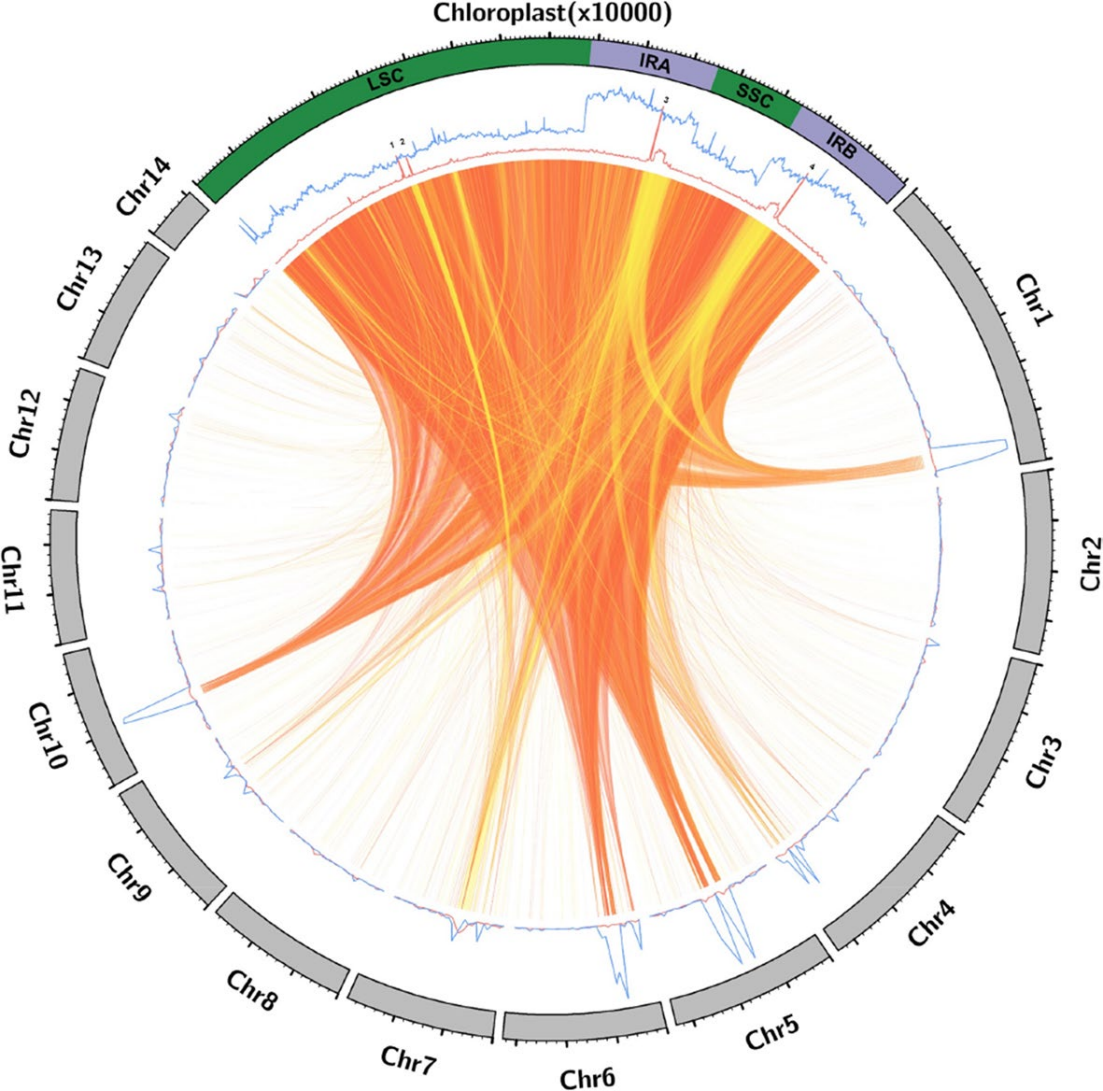
Circos plot representation of NUPTs in the moringa nuclear genome. Nuclear and chloroplast chromosomes are represented as grey and green filled blocks, respectively, forming a circumference. Results are shown for the 14 nuclear chromosomes, hosting 4812 NUPTs (92.49% of the total number) spanning 8,928,478 bp (91.28% of the total length). The block corresponding to the chloroplast genome is located at 12 o’clock, and the 14 nuclear chromosomes are arranged clockwise. Nuclear chromosomes are drawn to scale, with lengths proportional to size and expressed in Mb, while the chloroplast genome has been upscaled to occupy a quarter of the image circumference; its size unit was set to 10,000 bp. Line plots representing the respective density distributions of NUPTs-I (red) and NUPTs-II (blue) are displayed. Windows of 500,000 and 100 bp were selected for the nuclear and chloroplast chromosomes, respectively. Local BLASTN sequence alignments between the chloroplast and the nuclear genome corresponding to individual NUPTs are represented as ribbons. Ribbons are colored according to the percentage of sequence identity of the local alignments (NUPTs) grouped by quartiles (with yellow, light orange, orange, and red corresponding to the first, second, third and fourth quartiles, respectively). LSC, Large Single Copy; IRA, Inverted Repeat A; IRB, Inverted Repeat B; SSC, Small Single Copy

The distribution and frequency of NUPTs across the 14 chromosomes conforming the moringa nuclear genome was represented in a Circos plot as independent density plots for every episode (Fig. 2). In contrast to NUPTs-I, which showed an apparent homogenous distribution throughout the moringa nuclear genome, most NUPTs-II appeared to be highly concentrated in some specific regions of chromosomes one, four, five, six and 10, which showed prominent peaks in the density plots, likely corresponding to hotspots where NUPTs integration and / or subsequent fixation is favored (Fig. 2).

A recent survey in African and Asian rice reported a compositional bias at the flanking regions of NUPTs’ insertion sites [22]. Similarly, we examined whether the 100 bp regions flanking regions of NUPTs in moringa also showed any compositional bias. While the 100 bp flanking regions of NUPTs-I were featured by a greater GC content on average (36.4%) than the rest of the genome after excluding NUPT sequences (35.72%), the opposite trend was observed for NUPTs-II, which displayed a lower GC content on average (32.3%) with differences being significant according to Mann-Whitney U-tests (*P* = 2.07 × 10^−14^; *P* = 2.99 × 10^−103^, respectively).

Moreover, previous analysis on NUPTs from Arabidopsis and rice identified their tendency to group in clusters, defined as a group of two or more non-overlapping NUPTs where the distance between two consecutive integrants was less than 5 kb [7]. We tried to determine whether NUPTs in moringa were also forming clusters. 880 NUPTs (16.91% of the total) summing up a total of 1,232,888 bp (12.6% of the total) were found grouping into 282 clusters, which were detected in the 14 chromosomes plus nine scaffolds, and whose sizes ranged from 122 to 46,929 bp **(**Supplemental Table S4).

**Fig. 3:**
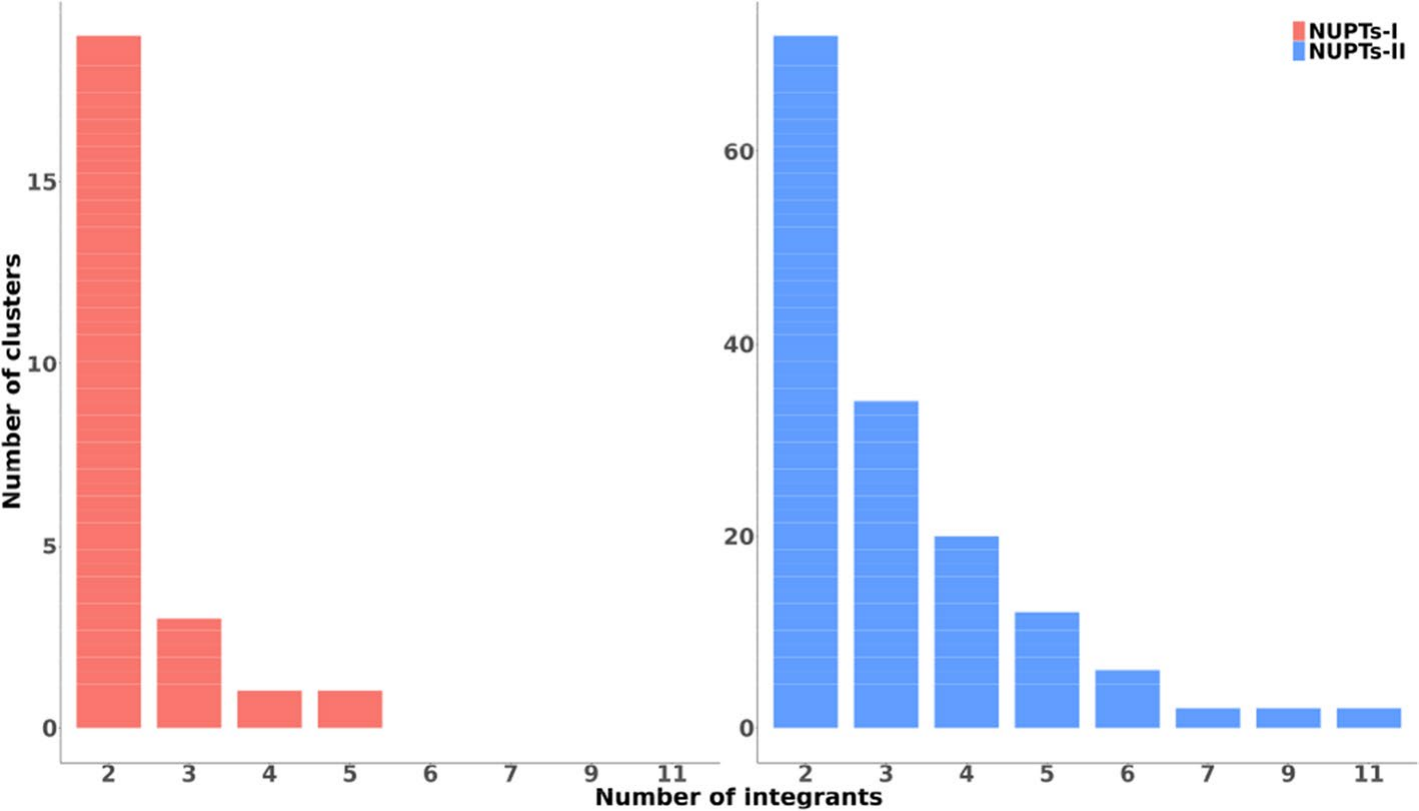
Distribution of the number of integrants of NUPT clusters

Then we examined separately clusters grouping NUPTs from every episode. 56 NUPTs-I (i.e., 7.22%) summing up a total of 18,145 bp (i.e., 7.17%) were found forming 24 clusters which hosted up to five integrants (Fig. 3) (Supplemental Table S4), whereas 476 NUPTs-II (i.e., 12.35%) summing up a total of 976,761 bp (i.e., 10.63%) were found inside 150 clusters which hosted up to 11 integrants (Fig. 3) (Supplemental Table S4). The rest of the clusters (108) hosted 380 NUPTs from either one or both episodes and / or unclassified NUPTs (Supplemental Table S4).

**Fig. 4:**
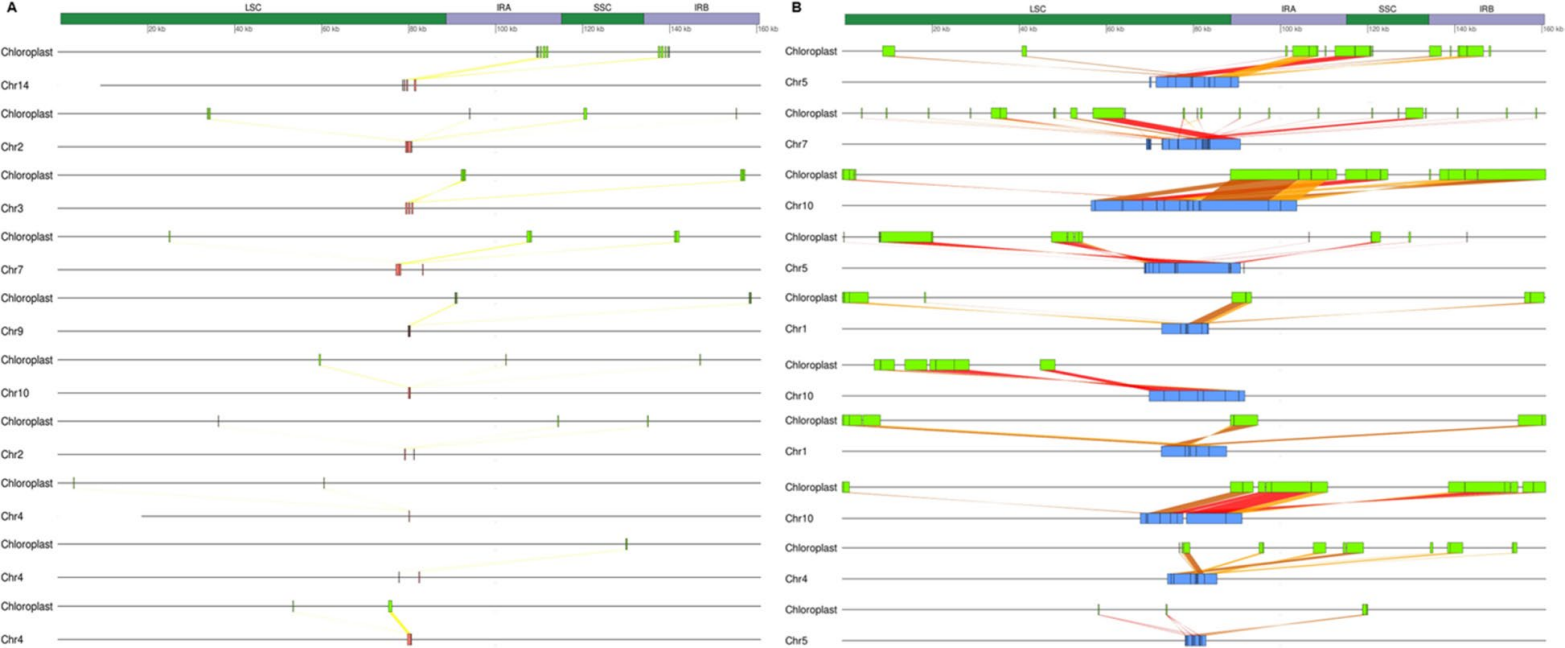
Graphical representation of the ten largest clusters of NUPTs for every episode in terms of number of integrants and the corresponding donor regions in the chloroplast genome (**A**) NUPTs-I. **B** NUPTs-II. For every cluster, donor regions in the chloroplast genome are shown as green blocks, while NUPTs-I and NUPTs-II are depicted as red and blue blocks, respectively. For every NUPT, the corresponding BLASTN sequence alignment between the chloroplast and the nuclear genome is represented as a ribbon. Ribbons are colored according to the percentage of sequence identity of the underlying alignment grouped by quartiles (with yellow, light orange, orange, and red corresponding to the first, second, third and fourth quartiles, respectively). The different elements in the diagram are drawn to scale, with the chloroplast genome and its four canonical regions (LSC, Large Single Copy; IRA, Inverted Repeat A; IRB, Inverted Repeat B; SSC, Small Single Copy) displayed on top as a reference for size

We further checked whether the ordering of NUPTs within individual clusters were arranged collinearly with respect to the chloroplast genome or were rather shuffled in some way. For this purpose, we graphically represented the ten largest clusters in terms of number of integrants from every episode and the corresponding donor regions in the chloroplast genome (Fig. 4). While clusters formed by NUPTs-I showed a tendency to be arranged collinearly with the chloroplast genome (Fig. 4A), no such collinearity could be observed for clusters of NUPTs-II (Fig. 4B).

The grouping into clusters of NUPTs at specific positions might be reflecting either large NUPTs fragmenting over time after their integration into the nuclear genome or chromosomal hotspots. If the former were the case, the sequence identity of NUPTs should correlate with their tendency to group into clusters. To test this hypothesis, we examined the correlation between the average sequence identity of the NUPTs in every cluster and the number of integrants. The tests were performed independently on clusters formed exclusively by NUPTs-I and NUPTs-II. No significant correlation was found for NUPTs from either episode (Supplemental Table S5).

### Biased distribution of NUPTs-I in the moringa chloroplast genome

Finally, we studied the distribution of NUPTs across the moringa chloroplast genome. For this purpose, we divided the corresponding DNA sequence into 100 bp regions and represented the frequency of occurrence of NUPTs as density plots (Fig. 2). We performed the analysis considering separately NUPTs-I and NUPTs-II. From the density plots of NUPTs-I, four peaks were apparent, which accounted for 354 NUPTs-I, i.e., 45.61% of the total. Two of the peaks, designated 1 and 2 (Fig. 2), spanned 200 bp each and were located in almost consecutive regions of the Large Single Copy (LSC) region of the chloroplast genome. The remaining two, designated 3 and 4 (Fig. 2), were of 3800–3900 bp in size and corresponded to redundant sequences from the IR regions of the chloroplast genome. In contrast, NUPTs-II were found to be almost uniformly distributed across the chloroplast genome, except for the IR regions, where, as expected, around twice the number of NUPTS-II could be observed (Fig. 2).

## Discussion

By leveraging a recently obtained high-quality long-read chromosome-scale assembly of the nuclear genome of moringa (i.e., AOCCv2) [23], we gained a finer characterization of the rich fraction of plastid DNA originally detected in an older, less contiguous, version (i.e., AOCCv1) [26], the highest reported for any plant species so far [19]. While the total fraction of plastid DNA was similar using both versions of the genome, differences were observed regarding the events underlying such enrichment. Our previous report [19], using the distribution of synonymous substitutions rates as a proxy of evolutionary time, attributed such enrichment in plastid DNA to a recent single burst of plastid gene duplicates relocating to the moringa nuclear genome. Here, in turn, by fitting Gaussian mixture models to the distributions of sequence identity of NUPTs (taken instead as a proxy of evolutionary time), two distinct main episodic events of NUPTs’ formation could be detected, namely NUPTs-I and NUPTs-II. The reason for this discrepancy likely resides in errors in the annotation of the AOCCv1 moringa nuclear genome, featured by an overrepresentation of small genes annotated with chloroplast and photosynthetic functions. While 656 and 114 genes were annotated with the terms “chloroplast” or “photosynthesis”, respectively, in the AOCCv1 moringa genome, only 378 and 51 genes were annotated with such terms in AOCCv2 [23]. For example, while 45 fragmented nuclear genes were annotated as encoding for the plastid-encoded large subunit of ribulose-1,5-bisphosphate carboxylase/oxygenase (RBCL) in AOCCv1, only three were annotated as such in AOCCv2, although all of them could be mapped to specific genomic regions in AOCCv2. Altogether suggests the previous enrichment in chloroplast related functions observed among nuclear genes was likely due to fragmented DNA of plastid origin, i.e., NUPTs, encompassing coding regions, wrongly annotated as gene coding models.

Hitherto, relative ages of NUPTs’ formation in different plant species had been reported to be featured by either exponentially decreasing or uniformly constant distributions [15, 17, 18], which fit, respectively, into two different modes of NUPTs´ formation, i.e., single events and hotspots [7, 28]. The single event mode commonly results in long continuous NUPTs collinear with specific regions of the chloroplast genome, which are concentrated in specific regions of the nuclear genome, e.g., (peri)centromeric regions [7, 15, 16, 28], and are expected to decay into smaller fragments and relocate as a consequence of chromosomal rearrangements and reshuffling involving transposable element activity [16]. In contrast, hotspots result in the concomitant integration of multiple short NUPTs from different origins arranged as a mosaic in specific loci of the nuclear genome [28, 29].

To the best of our knowledge, no previous studies have reported the bimodal distribution of NUPT relative ages observed here for moringa. The observed bimodal distribution implies NUPTs in moringa were formed through two events separated in time. Furthermore, NUPTs from every event showed markedly distinctive features, suggesting they originated through distinct mechanisms. For example, according to the relative distribution of sizes, younger NUPTs from episode II showed seemingly random origins throughout the chloroplast genome and were featured by a wide range of sizes, their preferential location in hotspots across the nuclear genome and negative correlation between sequence identity and size. However, although some NUPTs-II may have originated as long fragments subsequently breaking into smaller pieces arranged collinearly as clusters throughout the nuclear genome, in accordance with the single event mode [28], no correlation was observed between the number of NUPTs-II grouping in clusters and sequence identity. This lack of correlation suggests at least some NUPTs-II may have also originated as smaller fragments landing in specific landmarks of the nuclear genome, i.e., chromosomal hotspots, eventually further dispersing trough different kinds of genome rearrangements. This was also in agreement with the observation that NUPTs-II grouped in clusters tended to be found shuffled in some way rather than arranged collinearly with the chloroplast genome. Altogether supports the origin of NUPTs-II through both single events and hotspots modes of origin.

In turn, older NUPTs from episode I, featured by a narrower distribution of sizes, no correlation between sequence identity and size and a tendency to be arranged colinearly with the chloroplast genome when found grouped in clusters, do not seem to fit into any of the two modes of NUPTs’ formation previously described. Moreover, almost half of the NUPTs from episode I originated from four specific regions in the chloroplast genome, an observation only reported previously for *Asparagus officialis* [20] and in contrast to previous studies in Arabidopsis, rice and other species, which showed a homogenous distribution of NUPTs throughout the chloroplast genome [15, 17]. We therefore propose here a third mode of NUPTs’ formation through small-scale recurrent events. Once individual NUPTs are formed, two scenarios are plausible i) multiple copies of NUPTs firstly forming in the chloroplast and later relocating to the nucleus, or ii) individual NUPTs recurrently duplicating once integrated into the nuclear genome.

In respect of the possible evolutionary forces underlying the leakages and subsequent fixation of variable amounts of plastid DNA in plant nuclear genomes, these might be related to the different stressful conditions to which every species would have been subjected throughout their recent evolutionary history; different stresses have been shown to promote DNA migration from chloroplasts to the nucleus [10, 30]. The massive amounts of plastid DNA found in the moringa nuclear genome might be well related to the exposure to stressful conditions during its recent evolutionary history [31, 32]. Indeed, domestication of moringa from the sub-Himalayan lowlands in NW India, its putative location of origin where mean annual precipitations exceed 1100 mm, to tropical and sub-tropical areas around the world where its culture has spread [31] likely involved the selection of varieties better adapted to drier and hotter environments [32, 33]. Furthermore, moringa shows a great adaptive potential to successfully cope with multiple stresses, particularly water deficit and UVB radiation [34]. At this respect, it has been noted that the 11 giant NUPTs found in Asian rice trended to distribute in natural populations from higher latitude regions featured by lower temperatures and light intensities [22]. This observation led the authors to attribute NUPTs a potential role in enhancing environmental adaptation by increasing the number of chloroplast-derived genes which might, in turn, improve photosynthesis [22]. However, we believe this adaptive-to-stress hypothesis seems unlikely given that “recent” plastid-to-nuclear gene transfers are exceedingly rare, especially for photosynthetic genes, with the genes most frequently transferred in extant lineages being ribosomal proteins [35]. Whatever the specific forces that are at the origin of the fixation of the massive amounts of plastid DNA found in the moringa nuclear genome, they appear to be of a different nature for every independent event of NUPTs formation detected here.

## Conclusions

Results presented here reveal an unanticipated complexity of the mechanisms at the origin of NUPTs and of the evolutionary forces behind their fixation. Comparative genomics of domesticated moringa together with that of the 12 wild *Moringa* species that make up the taxonomic family Moringaceae within the Brassicales order [36], emerges as an excellent model for reconstructing the mechanisms of origin and evolutionary fixation of plastid DNA in the nuclear genome.

## Methods

### Detection and analysis of plastid DNA in the nuclear genome

NUPTs in the published versions of the moringa nuclear genome [25–27] were detected using the BLASTN local alignment tool from the BLAST+ program package v2.12.0+ [37]. The chloroplast genome sequence of moringa [24] (Table 1) was used as query and the published versions of its nuclear genome sequence (Table 1) as databases. The parameters were as follows: -evalue 1e-5 -word_size 9 -penalty − 2 -show_gis -dust no -num_threads 8. In order to deal with low complexity regions putatively present in the chloroplast genome that might result in spurious alignments wrongly detected as homologous regions, the analyses were repeated by turning on the -dust setting (−dust yes). Results in terms of sequence identity and density of NUPTs were represented as circular plots, constructed using Circos version 0.69–8 [38]. In order to correct for redundancy of NUPTs resulting from the IR region of the chloroplast genome, BLASTN hits involving IR regions were counted only once.

In order to detect NUPTs showing 100% identity with the chloroplast genome plus their 100 bp flanking regions in the previous published versions of the moringa nuclear genome, BLASTN alignments were firstly performed using the whole set of 100% identity NUPTs as query and the genome sequence of each version as database. NUPTs and their best scoring hits detected in each version of the genome were then aligned using the MUSCLE algorithm [39] through the SeaView v5.0.5 program [40]. The resulting multiple sequence alignments were edited using GeneDoc v2.7 [41].

In order to examine whether NUPTs in clusters were arranged collinearly with the donor regions of the chloroplast genome or shuffled in some way, the corresponding BLASTN alignments were visualized through the R genoPlotR v 0.8.11 package [42].

### Gaussian mixture modeling of NUPTs’ percent identity distribution

In order to detect peaks in the distribution of percent identity values putatively corresponding to episodic events of NUPTs integration in the nuclear genome, Gaussian mixture models were fitted to the corresponding distribution by employing the Expectation-Maximization (EM) algorithm for mixtures of normal distributions. We first determined the optimal number of Gaussian components (k) using the boot.comp() function from the R mixtools v1.2 package [43], which performs a parametric bootstrap by producing B bootstrap realizations (replicates) of the likelihood ratio statistic for testing the null hypothesis of a k-component fit versus the alternative hypothesis of a (k + 1)-component fit to various mixture models. For this step, we used 1000 replicates, a significance level of 0.01, and set the maximum number of components to nine. The number of components determined in the previous step was then used to fit a mixture of Gaussian models to the distribution of percent identity values, utilizing the normalmixEM() function from the same package and the following parameters: maxit = 1e-30, maxrestarts = 1e− 3, epsilon = 1e− 10. Each peak was characterized by an age (expressed in percent identity values) that corresponded to the mean of the Gaussian mixture component. Several other parameters were estimated from each of the models, including the standard deviation of each component, as well as the mixing probabilities of each NUPT of belonging to each retrieved peak.

### Supplementary Information

**Additional file 1.**

**Additional file 2.**

**Additional file 3.**

**Additional file 4.**

**Additional file 5.**

**Additional file 6.**

**Additional file 7.**

**Supplemental Tables.**

**Supplemental Figures.**

## Data Availability

All data generated or analyzed during this study are included in this article and its Supplemental information files.

## Abbreviations

NUPT: nuclear plastid DNA sequence
DSB: double-stranded break
NHEJ: non-homologous end joining
SSA: single strand annealing
