# Supplementary material for: Spatial and temporal characterization of the rich fraction of plastid DNA present in the nuclear genome of Moringa oleifera reveals unanticipated complexity in NUPTs´ formation
Source: BMC Genomics. 2024 Jan 15;25:60. doi: 10.1186/s12864-024-09979-5 (PMC10789010; doi:10.1186/s12864-024-09979-5)
Supplement: Supplementary file 1 — Additional file 1. [file 12864_2024_9979_MOESM1_ESM.docx]

**Additional file 1**. Sequences in FASTA format corresponding to two putative low complexity regions found in the moringa chloroplast genome.

>NC_041432.1:8600-8800

TTTTTATTTACATTTATTAAAATAAAAGAAAATCTTAATTATAATATATTAATAATATAATATATCTTTTTATTATATAATATATCTTTTTATTATTTATTATTTATTTTTATATTATATTATTAATTTTTATTTTATAATAAAATTAATTTTTATTTTATAATAAATTATTTTATAAGAAATTTTATTATATTTTTTTT

>NC_041432.1:53800-54150

TTATCTATTTTCATTATTTTTATTTTATTATTATATTCATTTTTATTTATTTTATTATTATATTATATTAAATTATTTATATTAAATTATTTATATTAAACATTTTTATTTATTTTATTTTTATTTATTTTATTATTATATTATATTAATAATATTATTTTATTATATTAATAATATTATTTTAATATTAATTATTTTAATATTTAATATTAATTATTTTAATATTAATTATTTTTTATTTTTAATTTTAAAATAAAATTATTAAATTAAAATTATTAAATAATAAATAAAAAATAAAATTTTTTTTATTATTTATATTTATATTTATATTATAAAAATAAAATATTATGT
