# Supplementary material for: Spatial and temporal characterization of the rich fraction of plastid DNA present in the nuclear genome of Moringa oleifera reveals unanticipated complexity in NUPTs´ formation
Source: BMC Genomics. 2024 Jan 15;25:60. doi: 10.1186/s12864-024-09979-5 (PMC10789010; doi:10.1186/s12864-024-09979-5)

**Additional file 2**. **Multiple sequence alignment of NUPT showing 100% identity with the chloroplast genome plus 100 bp flanking regions in four different versions of the moringa nuclear genome.**

Chr2: 14347497-14347732

JAJFZO010000718.1: 547581-547816

Scaffold36150: 892679-892914

Scaffold103: 235471-235706


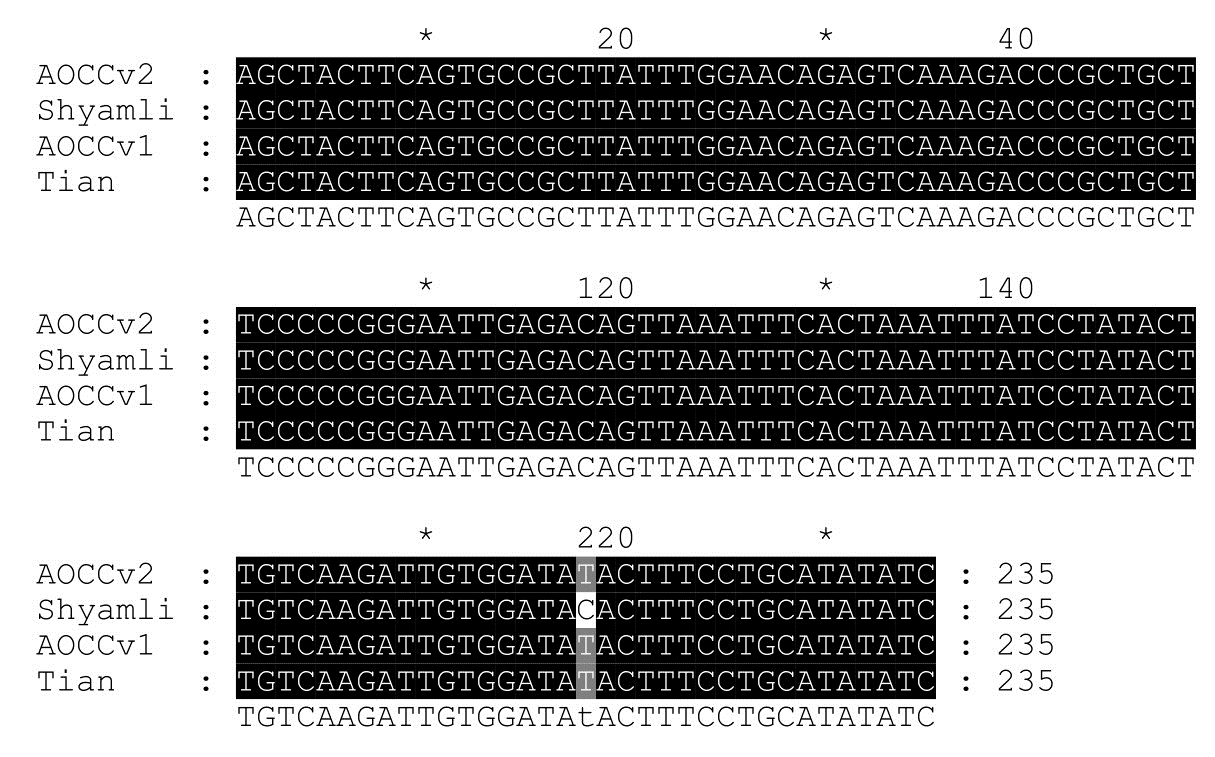

Supplement: Supplementary file 2 — Additional file 2. [file 12864_2024_9979_MOESM2_ESM.docx]
