# Supplementary material for: Spatial and temporal characterization of the rich fraction of plastid DNA present in the nuclear genome of Moringa oleifera reveals unanticipated complexity in NUPTs´ formation
Source: BMC Genomics. 2024 Jan 15;25:60. doi: 10.1186/s12864-024-09979-5 (PMC10789010; doi:10.1186/s12864-024-09979-5)

**Additional file 3**. **Multiple sequence alignment of NUPT showing 100% identity with the chloroplast genome plus 100 bp flanking regions in four different versions of the moringa nuclear genome.**

Chr1: 5591696-5591939

JAJFZO010000799.1: 2592415-2592658

Scaffold36466: 105118-105361

Scaffold454: 32192-32436


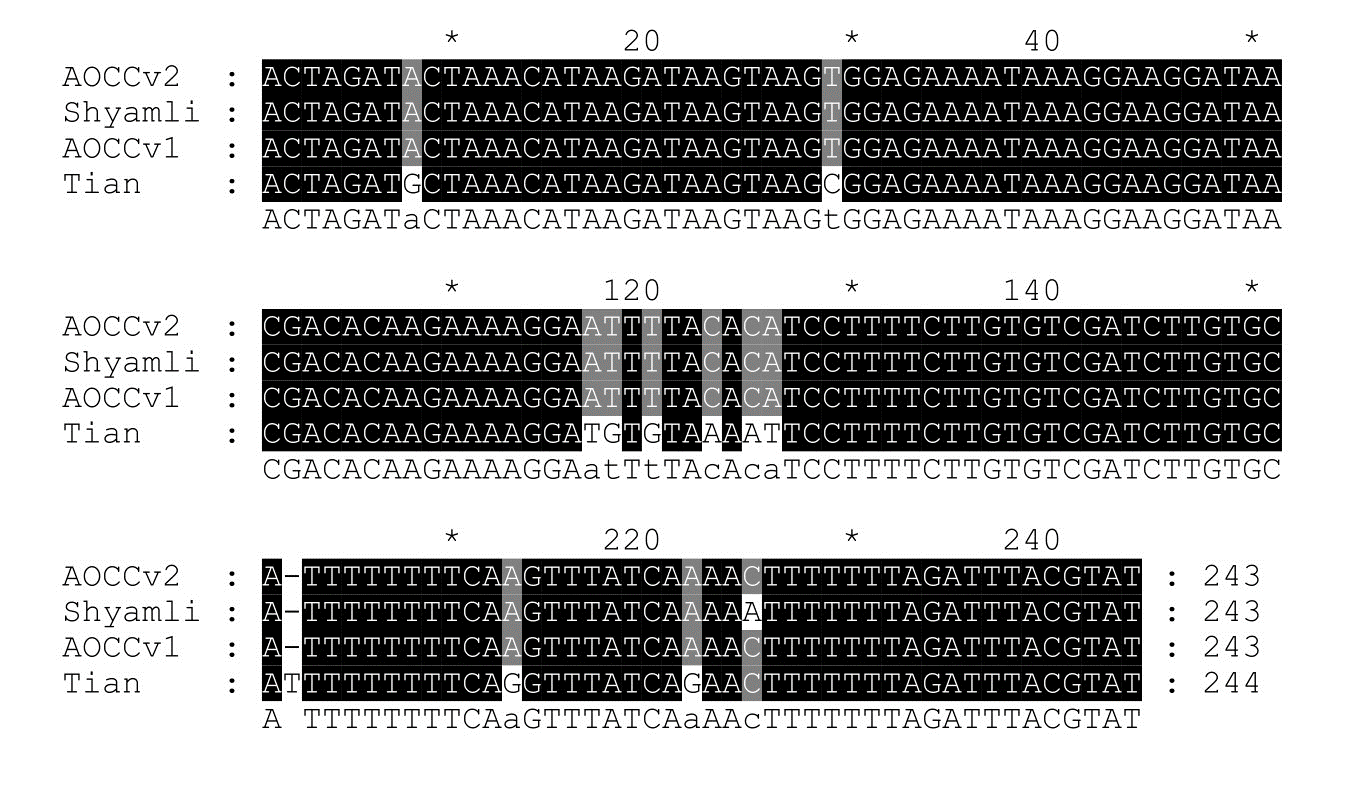

Supplement: Supplementary file 3 — Additional file 3. [file 12864_2024_9979_MOESM3_ESM.docx]
