# Supplementary material for: Spatial and temporal characterization of the rich fraction of plastid DNA present in the nuclear genome of Moringa oleifera reveals unanticipated complexity in NUPTs´ formation
Source: BMC Genomics. 2024 Jan 15;25:60. doi: 10.1186/s12864-024-09979-5 (PMC10789010; doi:10.1186/s12864-024-09979-5)

**Additional file 4**. **Multiple sequence alignment of NUPT showing 100% identity with the chloroplast genome plus 100 bp flanking regions in four different versions of the moringa nuclear genome.**

Chr4: 16350967-16351236

JAJFZO010000028.1: 2525455-2525186

Scaffold36239: 303572-303303

Scaffold162: 140414-140145


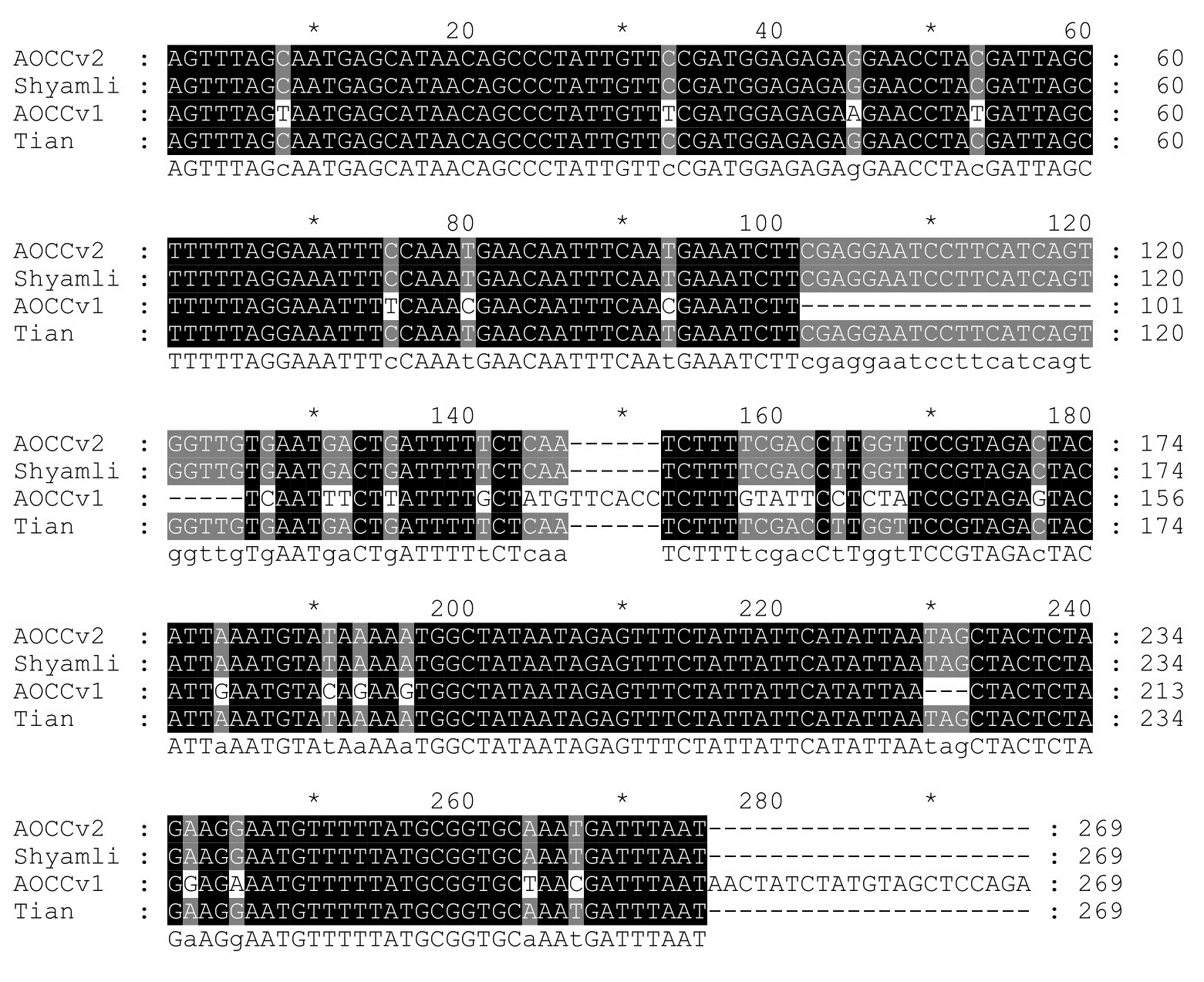

Supplement: Supplementary file 4 — Additional file 4. [file 12864_2024_9979_MOESM4_ESM.docx]
