# Supplementary material for: Spatial and temporal characterization of the rich fraction of plastid DNA present in the nuclear genome of Moringa oleifera reveals unanticipated complexity in NUPTs´ formation
Source: BMC Genomics. 2024 Jan 15;25:60. doi: 10.1186/s12864-024-09979-5 (PMC10789010; doi:10.1186/s12864-024-09979-5)

**Additional file 5**. **Multiple sequence alignment of NUPT showing 100% identity with the chloroplast genome plus 100 bp flanking regions in four different versions of the moringa nuclear genome.**

Chr6: 5371309-5371575

JAJFZO010000609.1: 4792427-4792693

Scaffold36351: 20141-20407

Scaffold276: 84952-84686


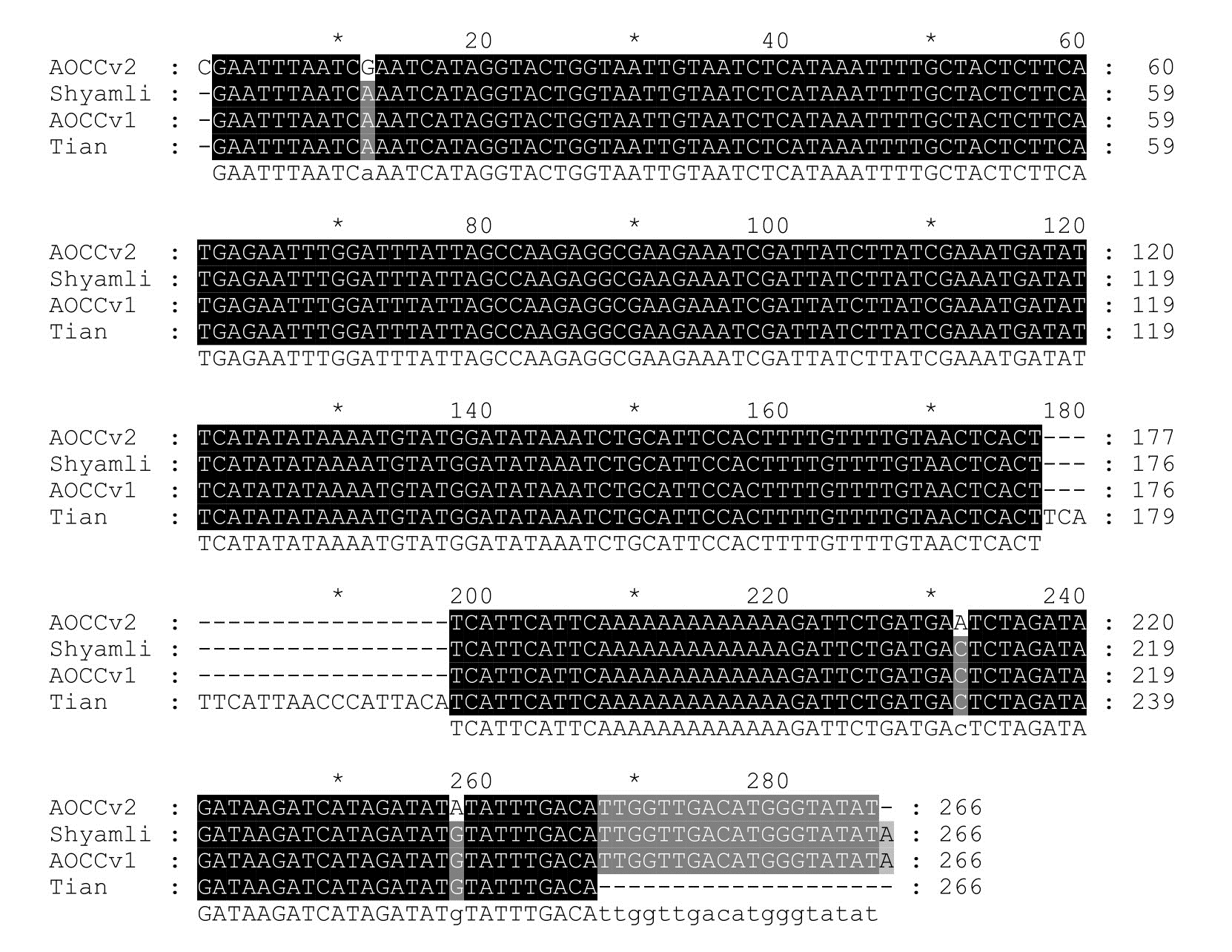

Supplement: Supplementary file 5 — Additional file 5. [file 12864_2024_9979_MOESM5_ESM.docx]
