# Supplementary material for: Spatial and temporal characterization of the rich fraction of plastid DNA present in the nuclear genome of Moringa oleifera reveals unanticipated complexity in NUPTs´ formation
Source: BMC Genomics. 2024 Jan 15;25:60. doi: 10.1186/s12864-024-09979-5 (PMC10789010; doi:10.1186/s12864-024-09979-5)

**Additional file 6**. **Multiple sequence alignment of NUPT showing 100% identity with the chloroplast genome plus 100 bp flanking regions in four different versions of the moringa nuclear genome.**

Chr14: 4851301- 4851611

JAJFZO010000722.1: 82738-83048

Scaffold36276: 12922- 12612

Scaffold1360: 84-394


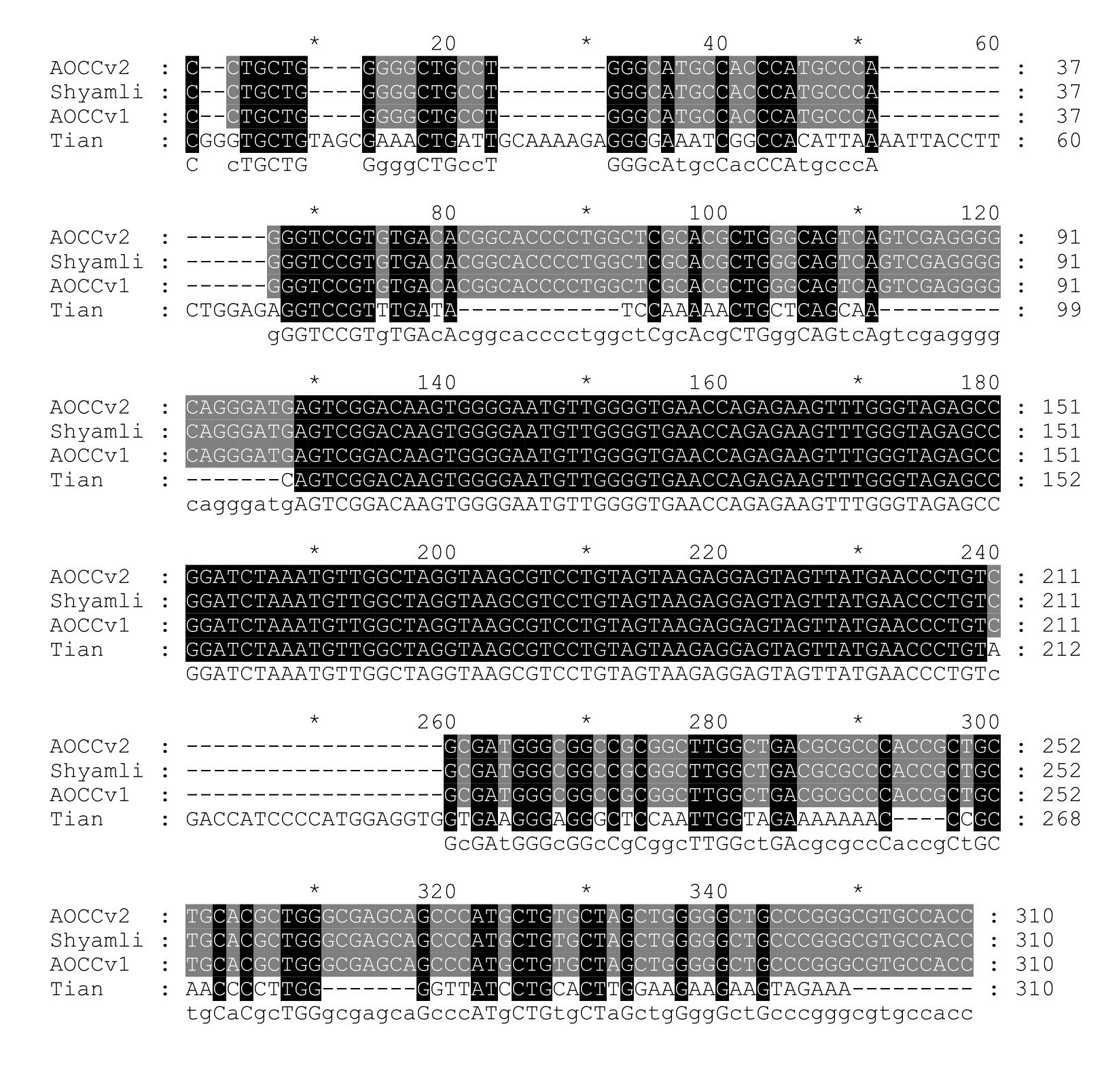

Supplement: Supplementary file 6 — Additional file 6. [file 12864_2024_9979_MOESM6_ESM.docx]
