# Supplementary material for: Spatial and temporal characterization of the rich fraction of plastid DNA present in the nuclear genome of Moringa oleifera reveals unanticipated complexity in NUPTs´ formation
Source: BMC Genomics. 2024 Jan 15;25:60. doi: 10.1186/s12864-024-09979-5 (PMC10789010; doi:10.1186/s12864-024-09979-5)

**Additional file 7**. **Multiple sequence alignment of NUPT showing 100% identity with the chloroplast genome plus 100 bp flanking regions in four different versions of the moringa nuclear genome.**

Chr5: 8111661-8113379

JAJFZO010000156.1: 18413-20131

Scaffold1113: 1307116-1305398

Scaffold50: 166105-167823


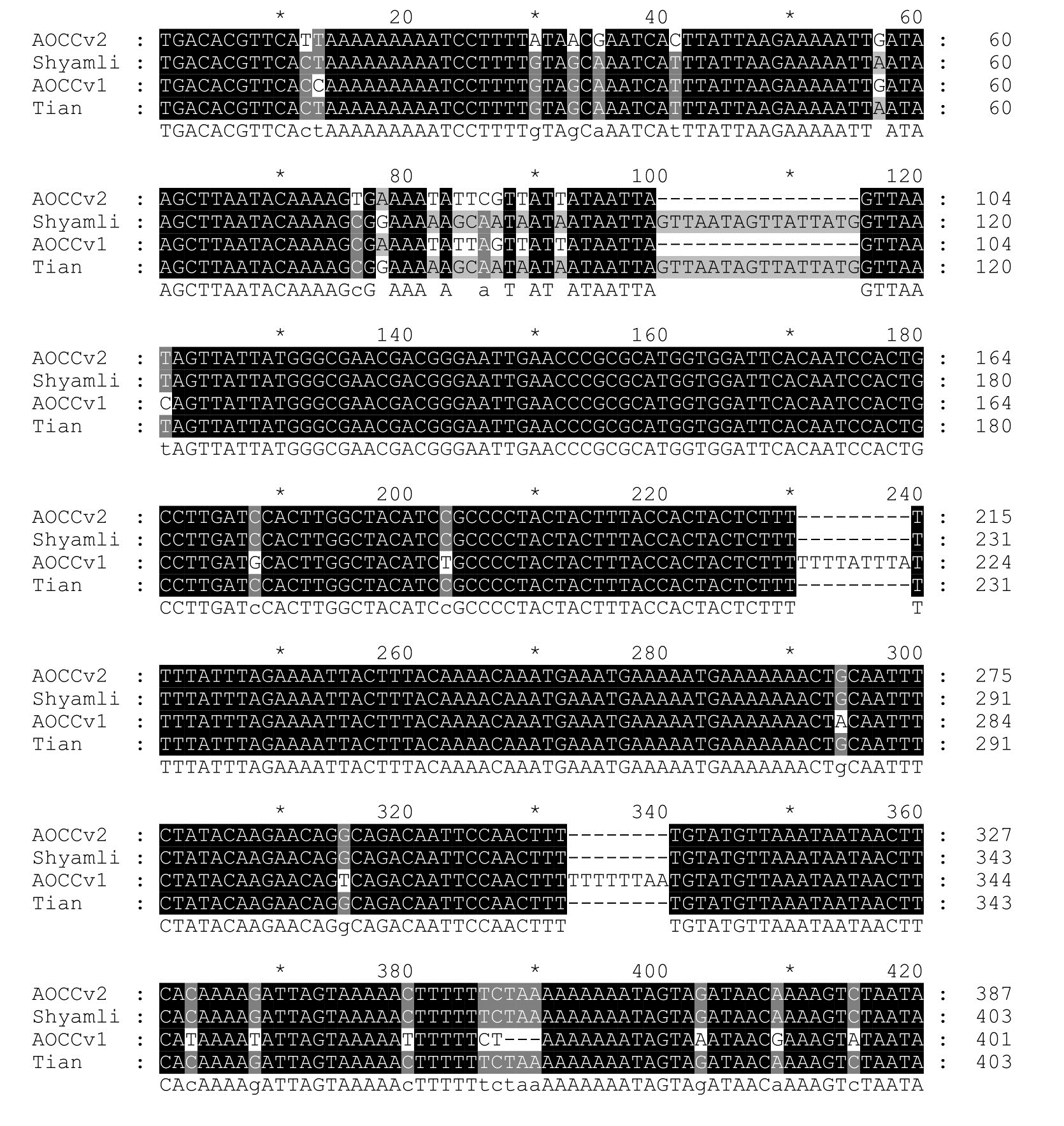


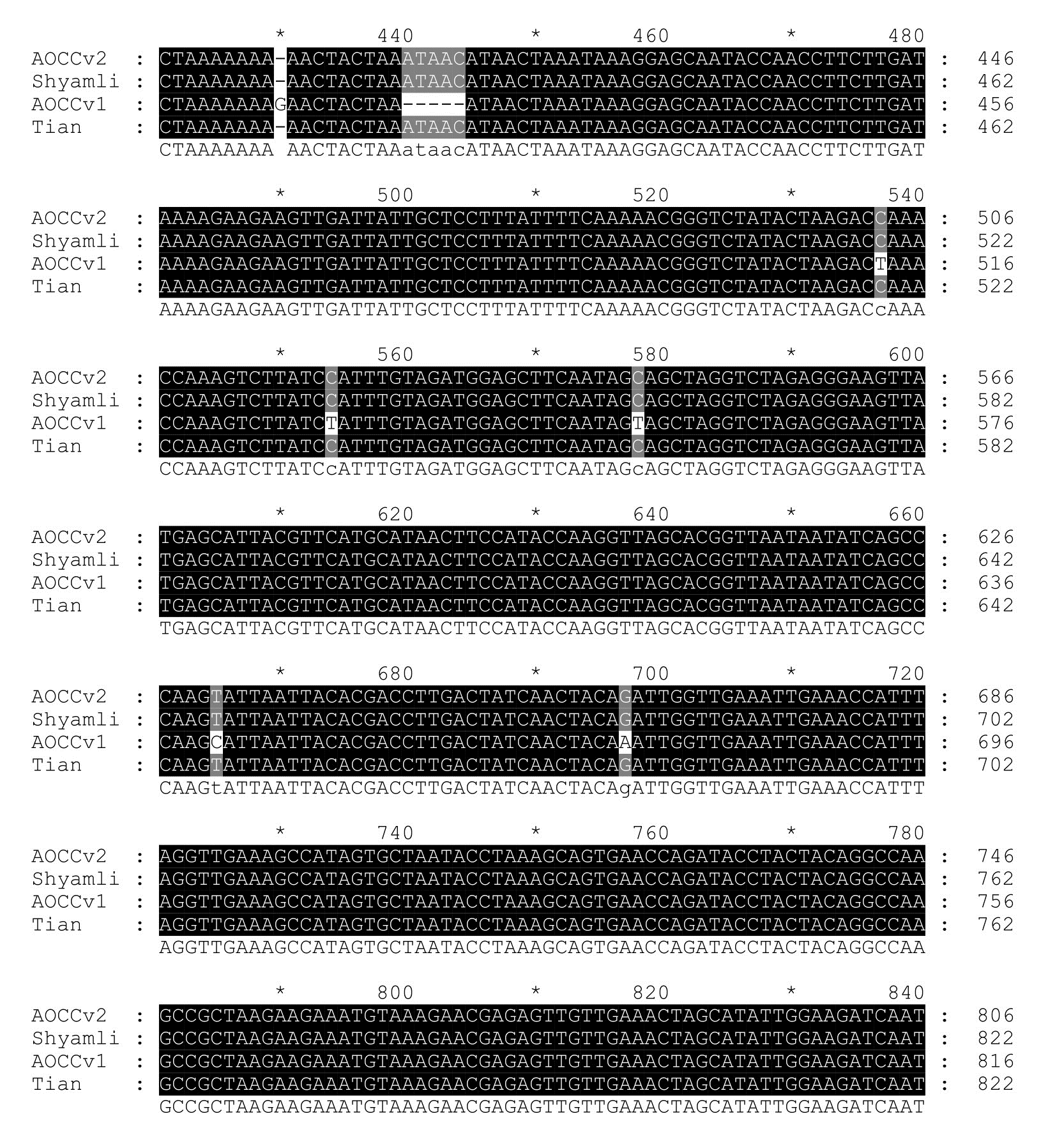


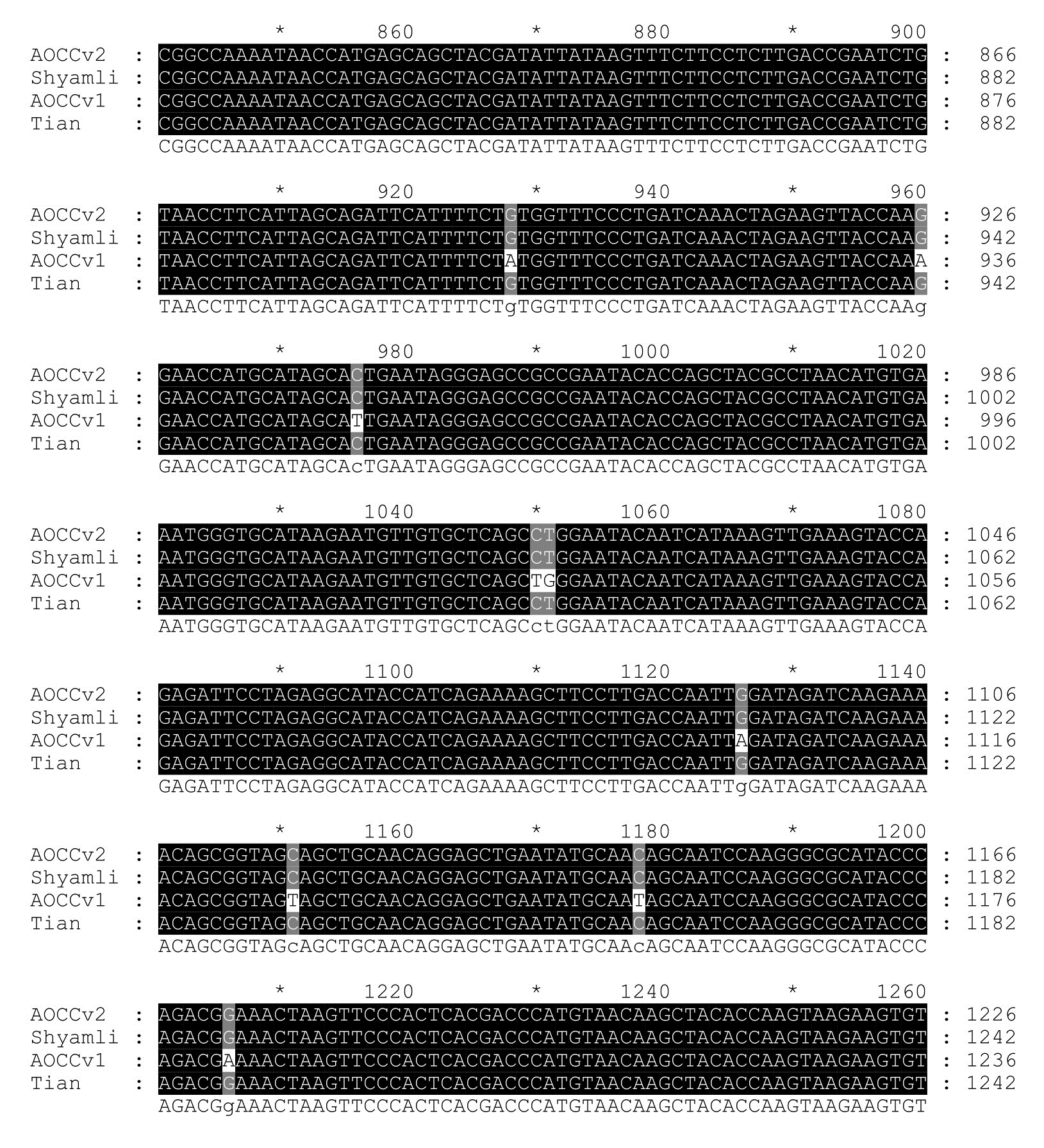


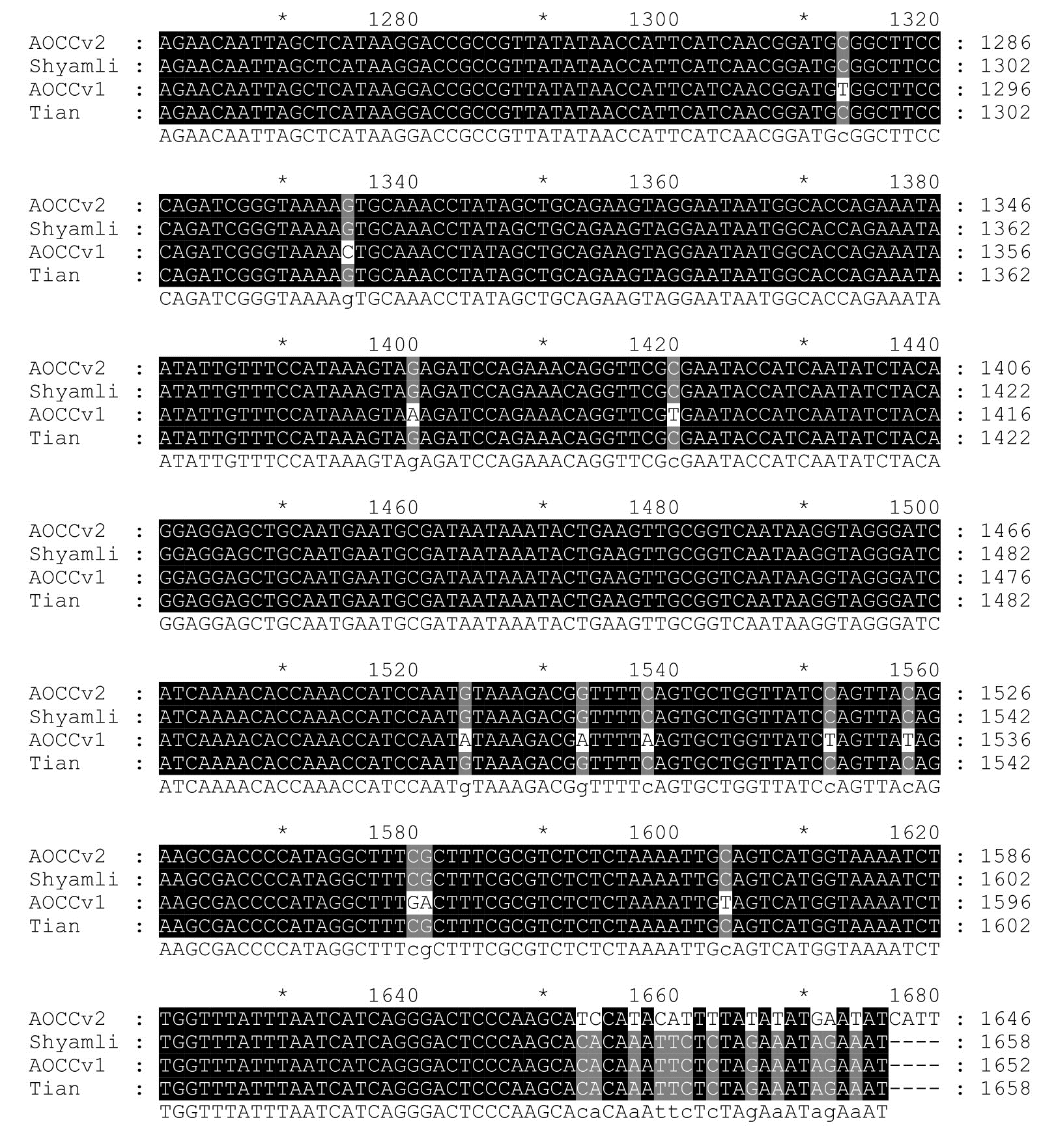


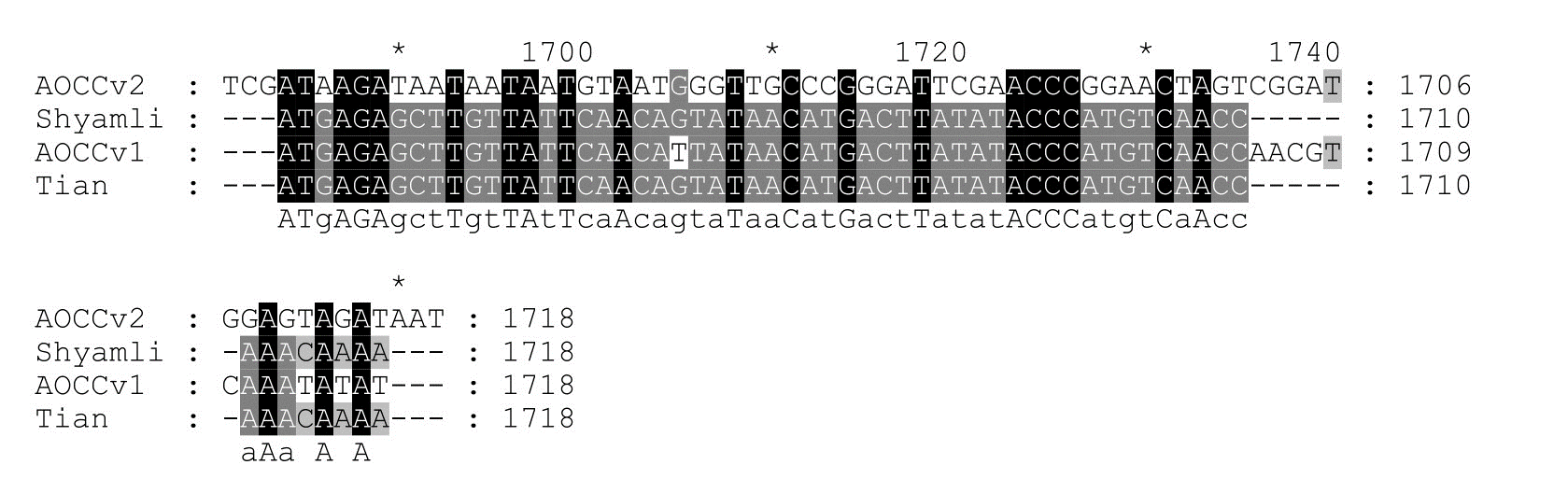

Supplement: Supplementary file 7 — Additional file 7. [file 12864_2024_9979_MOESM7_ESM.docx]
