# Supplementary material for: Spatial and temporal characterization of the rich fraction of plastid DNA present in the nuclear genome of Moringa oleifera reveals unanticipated complexity in NUPTs´ formation
Source: BMC Genomics. 2024 Jan 15;25:60. doi: 10.1186/s12864-024-09979-5 (PMC10789010; doi:10.1186/s12864-024-09979-5)
Supplement: Supplementary file 9 — Supplemental Figures. [file 12864_2024_9979_MOESM9_ESM.pptx]

## Slide 1
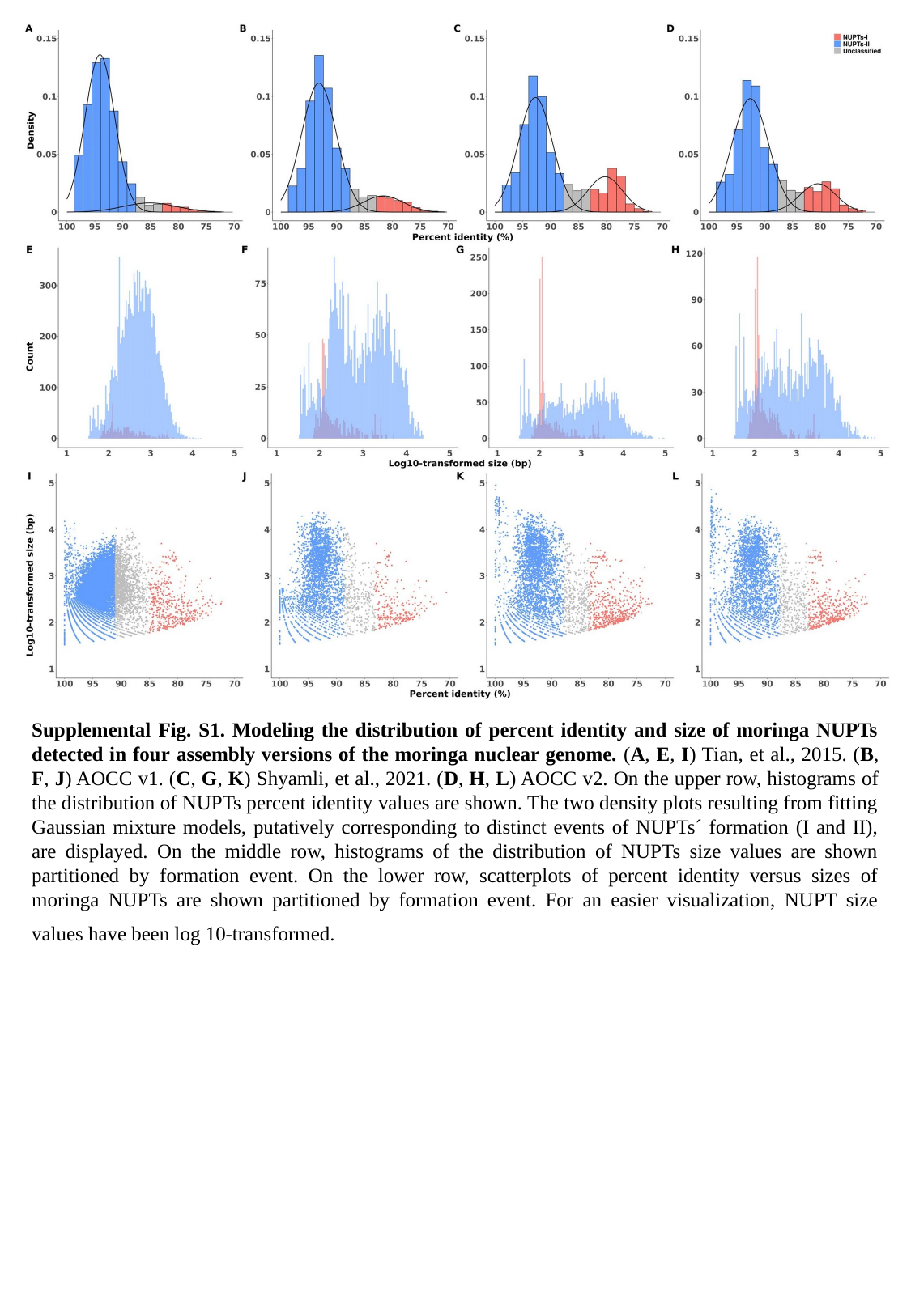

Supplemental Fig. S1. Modeling the distribution of percent identity and size of moringa NUPTs detected in four assembly versions of the moringa nuclear genome. (A, E, I) Tian, et al., 2015. (B, F, J) AOCC v1. (C, G, K) Shyamli, et al., 2021. (D, H, L) AOCC v2. On the upper row, histograms of the distribution of NUPTs percent identity values are shown. The two density plots resulting from fitting Gaussian mixture models, putatively corresponding to distinct events of NUPTs´ formation (I and II), are displayed. On the middle row, histograms of the distribution of NUPTs size values are shown partitioned by formation event. On the lower row, scatterplots of percent identity versus sizes of moringa NUPTs are shown partitioned by formation event. For an easier visualization, NUPT size values have been log 10-transformed.
